# Crystal structure of 3-methyl-2,6-bis­(4-methyl-1,3-thia­zol-5-yl)piperidin-4-one

**DOI:** 10.1107/S1600536814018856

**Published:** 2014-08-30

**Authors:** A. Manimaran, K. Sethusankar, S. Ganesan, S. Ananthan

**Affiliations:** a Orchid Chemicals & Pharmaceuticals Linmited, Research & Developement Centre, Sozhanganallur, Chennai 600 119, India; b Department of Physics, RKM Vivekananda College (Autonomous), Chennai 600 004, India; c Department of Chemistry, Presidency College (Autonomous), Chennai 600 025, India

**Keywords:** crystal structure, thia­zole, piperidine, zigzag chains

## Abstract

In the title compound, C_14_H_17_N_3_OS_2_, the central piperidinone ring adopts a chair conformation and the thia­zole rings are inclined to its mean plane by 80.16 (12) and 67.15 (12)°. The O atom and methyl group C atom deviate significantly from the mean plane of the central piperidinone ring, by 0.8138 (2) and 0.3175 (2) Å, respectively. The dihedral angle between the thia­zole rings is 51.88 (13)°. In the crystal, mol­ecules are linked *via* C—H⋯O hydrogen bonds, forming zigzag *C*(10) chains running parallel to [001].

## Related literature   

For biological and pharmaceutical applications of piperidino­nes and thia­zoles, see: Ganellin & Spickett (1965 ▶). For the synthesis of substituted piperidin-4-ones and their derivatives, see: Noller & Baliah (1948 ▶). For related structures, see: Gayathri *et al.* (2008 ▶); Nithya *et al.* (2009 ▶).
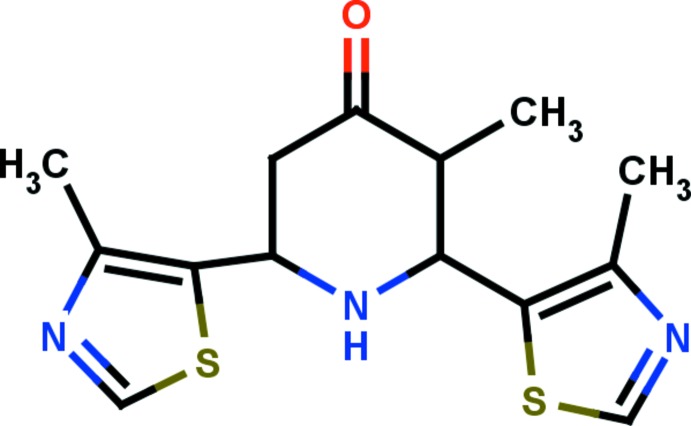


## Experimental   

### Crystal data  

C_14_H_17_N_3_OS_2_
*M* *_r_* = 307.43
Orthorhombic, 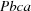

*a* = 11.389 (5) Å
*b* = 12.660 (5) Å
*c* = 21.667 (5) Å
*V* = 3124 (2) Å^3^
*Z* = 8
Mo *K*α radiation
μ = 0.34 mm^−1^
*T* = 296 K
0.30 × 0.25 × 0.20 mm

### Data collection  

Bruker Kappa APEXII CCD diffractometer
Absorption correction: multi-scan (*SADABS*; Bruker, 2008 ▶) *T*_min_ = 0.903, *T*_max_ = 0.934
19116 measured reflections
3762 independent reflections
2539 reflections with *I* > 2σ(*I*)
*R*_int_ = 0.034

### Refinement  

*R*[*F* ^2^ > 2σ(*F* ^2^)] = 0.048
*wR*(*F* ^2^) = 0.150
*S* = 1.00
3762 reflections
184 parameters
H-atom parameters constrained
Δρ_max_ = 0.52 e Å^−3^
Δρ_min_ = −0.47 e Å^−3^

### 

Data collection: *APEX2* (Bruker, 2008 ▶); cell refinement: *SAINT* (Bruker, 2008 ▶); data reduction: *SAINT*; program(s) used to solve structure: *SHELXS97* (Sheldrick, 2008 ▶); program(s) used to refine structure: *SHELXL97* (Sheldrick, 2008 ▶); molecular graphics: *ORTEP-3 for Windows* (Farrugia, 2012 ▶) and *Mercury* (Macrae *et al.*, 2008 ▶); software used to prepare material for publication: *SHELXL97* and *PLATON* (Spek, 2009 ▶).

## Supplementary Material

Crystal structure: contains datablock(s) global, I. DOI: 10.1107/S1600536814018856/su2768sup1.cif

Structure factors: contains datablock(s) I. DOI: 10.1107/S1600536814018856/su2768Isup2.hkl

Supporting information file. DOI: 10.1107/S1600536814018856/su2768Isup3.cml

**Fig. 1.**
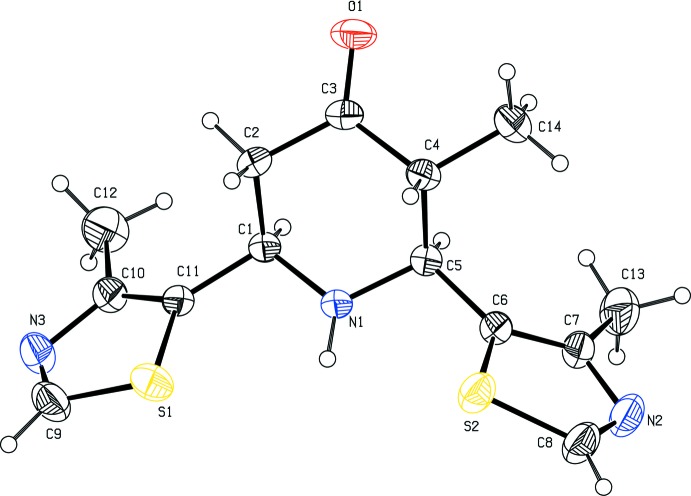
The molecular structure of the title molecular, with atom labelling. The displacement ellipsoids are drawn at the 30% probability level.

**Fig. 2.**
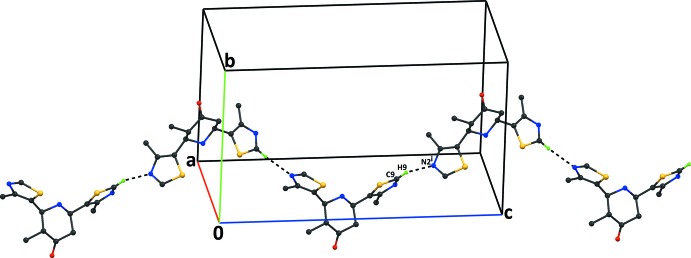
Part of the crystal packing of the title compound viewed along the b axis. Hydrogen bonds are shown as dashed lines; see Table 1 for details.

. DOI: 10.1107/S1600536814018856/su2768fig1.tif
The mol­ecular structure of the title mol­ecular, with atom labelling. The displacement ellipsoids are drawn at the 30% probability level.

b . DOI: 10.1107/S1600536814018856/su2768fig2.tif
Part of the crystal packing of the title compound viewed along the *b* axis. Hydrogen bonds are shown as dashed lines; see Table 1 for details.

CCDC reference: 1020191

Additional supporting information:  crystallographic information; 3D view; checkCIF report

## Figures and Tables

**Table 1:** Hydrogen-bond geometry (Å, °)

*D*—H⋯*A*	*D*—H	H⋯*A*	*D*⋯*A*	*D*—H⋯*A*
C9—H9⋯N2^i^	0.93	2.49	3.365 (4)	157

Symmetry code: (i) 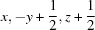.

## supplementary crystallographic information

### S1. Experimental

4-methyl-5-formyl thiazole (0.20 mol), 2-butanone (0.10 mol) and ammonium acetate (0.10 mol) were dissolved in 80 ml of distilled ethanol and heated over a boiling water bath with stirring for 8–10 h. Hydrochloric acid in isopropyl alcohol was added and the compound was filtered off as the hydrochloride salt under a nitrogen atmosphere. The compound was neutralized and extracted with dichloromethane. The dichloromethane layer was concentrated and crystals of the title compound were obtained by slow evaporation of a solution in ethanol.

### S2. Refinement

The H atoms were localized from difference electron density maps. During refinement they were treated as riding atoms: N-H = 0.86 Å, C—H = 0.93 - 0.98 Å with *U*_iso_(H) = 1.5*U*eq(C-methyl) and = 1.2U_eq_(N,C) for other H atoms.

### Crystal data

**Table:**

C_14_H_17_N_3_OS_2_	*F*(000) = 1296
*M**_r_* = 307.43	*D*_x_ = 1.307 Mg m^−^^3^
Orthorhombic, *P**b**c**a*	Mo *K*α radiation, λ = 0.71073 Å
Hall symbol: -P 2ac 2ab	Cell parameters from 2539 reflections
*a* = 11.389 (5) Å	θ = 2.6–28.4°
*b* = 12.660 (5) Å	µ = 0.34 mm^−^^1^
*c* = 21.667 (5) Å	*T* = 296 K
*V* = 3124 (2) Å^3^	Block, colourless
*Z* = 8	0.30 × 0.25 × 0.20 mm

### Data collection

**Table:**

Bruker Kappa APEXII CCD diffractometer	3762 independent reflections
Radiation source: fine-focus sealed tube	2539 reflections with *I* > 2σ(*I*)
Graphite monochromator	*R*_int_ = 0.034
ω and φ scans	θ_max_ = 28.4°, θ_min_ = 2.6°
Absorption correction: multi-scan (*SADABS*; Bruker, 2008)	*h* = −15→15
*T*_min_ = 0.903, *T*_max_ = 0.934	*k* = −16→14
19116 measured reflections	*l* = −27→21

### Refinement

**Table:**

Refinement on *F*^2^	Primary atom site location: structure-invariant direct methods
Least-squares matrix: full	Secondary atom site location: difference Fourier map
*R*[*F*^2^ > 2σ(*F*^2^)] = 0.048	Hydrogen site location: inferred from neighbouring sites
*wR*(*F*^2^) = 0.150	H-atom parameters constrained
*S* = 1.00	*w* = 1/[σ^2^(*F*_o_^2^) + (0.073*P*)^2^ + 1.5978*P*] where *P* = (*F*_o_^2^ + 2*F*_c_^2^)/3
3762 reflections	(Δ/σ)_max_ = 0.001
184 parameters	Δρ_max_ = 0.52 e Å^−^^3^
0 restraints	Δρ_min_ = −0.47 e Å^−^^3^

### Special details

**Table:**

Geometry. All e.s.d.'s (except the e.s.d. in the dihedral angle between two l.s. planes) are estimated using the full covariance matrix. The cell e.s.d.'s are taken into account individually in the estimation of e.s.d.'s in distances, angles and torsion angles; correlations between e.s.d.'s in cell parameters are only used when they are defined by crystal symmetry. An approximate (isotropic) treatment of cell e.s.d.'s is used for estimating e.s.d.'s involving l.s. planes.
Refinement. Refinement of *F*^2^ against ALL reflections. The weighted *R*-factor *wR* and goodness of fit *S* are based on *F*^2^, conventional *R*-factors *R* are based on *F*, with *F* set to zero for negative *F*^2^. The threshold expression of *F*^2^ > σ(*F*^2^) is used only for calculating *R*-factors(gt) *etc*. and is not relevant to the choice of reflections for refinement. *R*-factors based on *F*^2^ are statistically about twice as large as those based on *F*, and *R*- factors based on ALL data will be even larger.

### Fractional atomic coordinates and isotropic or equivalent isotropic displacement parameters (Å^2^)

**Table:**

	*x*	*y*	*z*	*U*_iso_*/*U*_eq_	
C1	0.2579 (2)	−0.01628 (17)	0.49282 (10)	0.0398 (5)	
H1	0.3299	−0.0482	0.4765	0.048*	
C2	0.1630 (2)	−0.10230 (18)	0.49779 (11)	0.0494 (6)	
H2A	0.1914	−0.1595	0.5235	0.059*	
H2B	0.0933	−0.0730	0.5170	0.059*	
C3	0.1325 (2)	−0.14405 (17)	0.43518 (11)	0.0447 (5)	
C4	0.1030 (2)	−0.06171 (17)	0.38720 (11)	0.0423 (5)	
H4	0.0300	−0.0275	0.4003	0.051*	
C5	0.19957 (19)	0.02385 (17)	0.38724 (10)	0.0393 (5)	
H5	0.2730	−0.0079	0.3727	0.047*	
C6	0.1691 (2)	0.11454 (18)	0.34598 (11)	0.0435 (5)	
C7	0.2132 (2)	0.1418 (2)	0.29028 (11)	0.0514 (6)	
C8	0.0832 (3)	0.2696 (2)	0.29990 (14)	0.0647 (8)	
H8	0.0403	0.3295	0.2895	0.078*	
C9	0.2859 (3)	0.1374 (3)	0.64598 (14)	0.0684 (8)	
H9	0.2725	0.1849	0.6780	0.082*	
C10	0.3678 (2)	0.0071 (2)	0.59575 (11)	0.0492 (6)	
C11	0.2849 (2)	0.03318 (17)	0.55366 (10)	0.0404 (5)	
C12	0.4537 (3)	−0.0816 (3)	0.59110 (16)	0.0757 (9)	
H12A	0.4317	−0.1368	0.6192	0.114*	
H12B	0.5307	−0.0564	0.6014	0.114*	
H12C	0.4538	−0.1087	0.5497	0.114*	
C13	0.3096 (3)	0.0875 (3)	0.25606 (15)	0.0752 (9)	
H13A	0.3797	0.1294	0.2583	0.113*	
H13B	0.2871	0.0787	0.2137	0.113*	
H13C	0.3239	0.0196	0.2742	0.113*	
C14	0.0807 (3)	−0.1090 (2)	0.32444 (12)	0.0630 (7)	
H14A	0.1513	−0.1420	0.3096	0.095*	
H14B	0.0573	−0.0544	0.2963	0.095*	
H14C	0.0195	−0.1609	0.3274	0.095*	
N1	0.21742 (16)	0.06327 (14)	0.44979 (8)	0.0402 (4)	
H1A	0.2050	0.1279	0.4602	0.048*	
N2	0.1627 (2)	0.2306 (2)	0.26420 (11)	0.0649 (6)	
N3	0.3680 (2)	0.0677 (2)	0.64846 (10)	0.0656 (6)	
O1	0.13353 (18)	−0.23686 (14)	0.42350 (9)	0.0653 (5)	
S1	0.20268 (6)	0.13746 (6)	0.58011 (3)	0.0607 (2)	
S2	0.06218 (6)	0.20349 (6)	0.36761 (3)	0.0582 (2)	

### Atomic displacement parameters (Å^2^)

**Table:**

	*U*^11^	*U*^22^	*U*^33^	*U*^12^	*U*^13^	*U*^23^
C1	0.0485 (12)	0.0321 (11)	0.0387 (12)	0.0025 (9)	−0.0018 (9)	−0.0007 (9)
C2	0.0679 (15)	0.0339 (11)	0.0464 (14)	−0.0065 (11)	−0.0023 (11)	0.0072 (10)
C3	0.0467 (12)	0.0337 (12)	0.0537 (14)	−0.0044 (9)	0.0014 (10)	−0.0022 (10)
C4	0.0451 (11)	0.0376 (11)	0.0443 (13)	−0.0024 (9)	−0.0015 (10)	−0.0042 (9)
C5	0.0441 (11)	0.0364 (11)	0.0375 (12)	−0.0004 (9)	−0.0007 (9)	−0.0003 (9)
C6	0.0492 (12)	0.0417 (12)	0.0397 (12)	−0.0049 (10)	−0.0033 (10)	0.0007 (9)
C7	0.0618 (15)	0.0530 (14)	0.0393 (14)	−0.0086 (12)	−0.0001 (11)	0.0052 (11)
C8	0.0773 (18)	0.0585 (17)	0.0583 (18)	0.0041 (14)	−0.0084 (15)	0.0211 (13)
C9	0.088 (2)	0.0675 (19)	0.0494 (17)	−0.0164 (17)	0.0147 (14)	−0.0199 (14)
C10	0.0536 (13)	0.0494 (14)	0.0446 (14)	−0.0061 (11)	−0.0038 (11)	−0.0004 (10)
C11	0.0491 (12)	0.0345 (11)	0.0377 (12)	−0.0008 (9)	0.0028 (9)	−0.0007 (9)
C12	0.0685 (18)	0.074 (2)	0.085 (2)	0.0149 (16)	−0.0254 (16)	−0.0040 (17)
C13	0.085 (2)	0.084 (2)	0.0569 (18)	−0.0011 (17)	0.0187 (15)	0.0051 (16)
C14	0.0793 (18)	0.0601 (17)	0.0496 (16)	−0.0130 (14)	−0.0044 (13)	−0.0115 (13)
N1	0.0541 (11)	0.0291 (9)	0.0376 (10)	0.0013 (8)	−0.0033 (8)	−0.0005 (7)
N2	0.0783 (15)	0.0663 (15)	0.0502 (14)	−0.0056 (13)	−0.0050 (12)	0.0214 (11)
N3	0.0822 (17)	0.0697 (16)	0.0449 (13)	−0.0143 (14)	−0.0069 (12)	−0.0080 (11)
O1	0.0847 (14)	0.0328 (9)	0.0784 (14)	−0.0010 (9)	−0.0094 (11)	−0.0062 (8)
S1	0.0656 (4)	0.0514 (4)	0.0651 (5)	0.0086 (3)	0.0065 (3)	−0.0160 (3)
S2	0.0670 (4)	0.0539 (4)	0.0538 (4)	0.0132 (3)	0.0032 (3)	0.0126 (3)

### Geometric parameters (Å, º)

**Table:**

C1—N1	1.448 (3)	C8—S2	1.706 (3)
C1—C11	1.491 (3)	C8—H8	0.9300
C1—C2	1.538 (3)	C9—N3	1.287 (4)
C1—H1	0.9800	C9—S1	1.713 (3)
C2—C3	1.497 (3)	C9—H9	0.9300
C2—H2A	0.9700	C10—C11	1.354 (3)
C2—H2B	0.9700	C10—N3	1.376 (3)
C3—O1	1.202 (3)	C10—C12	1.493 (4)
C3—C4	1.510 (3)	C11—S1	1.717 (2)
C4—C14	1.507 (3)	C12—H12A	0.9600
C4—C5	1.543 (3)	C12—H12B	0.9600
C4—H4	0.9800	C12—H12C	0.9600
C5—N1	1.458 (3)	C13—H13A	0.9600
C5—C6	1.496 (3)	C13—H13B	0.9600
C5—H5	0.9800	C13—H13C	0.9600
C6—C7	1.352 (3)	C14—H14A	0.9600
C6—S2	1.723 (3)	C14—H14B	0.9600
C7—N2	1.384 (3)	C14—H14C	0.9600
C7—C13	1.492 (4)	N1—H1A	0.8600
C8—N2	1.289 (4)		
			
N1—C1—C11	110.04 (18)	S2—C8—H8	122.4
N1—C1—C2	108.27 (18)	N3—C9—S1	115.9 (2)
C11—C1—C2	112.34 (19)	N3—C9—H9	122.0
N1—C1—H1	108.7	S1—C9—H9	122.0
C11—C1—H1	108.7	C11—C10—N3	115.1 (2)
C2—C1—H1	108.7	C11—C10—C12	126.5 (2)
C3—C2—C1	110.46 (19)	N3—C10—C12	118.3 (2)
C3—C2—H2A	109.6	C10—C11—C1	129.5 (2)
C1—C2—H2A	109.6	C10—C11—S1	110.05 (18)
C3—C2—H2B	109.6	C1—C11—S1	120.38 (17)
C1—C2—H2B	109.6	C10—C12—H12A	109.5
H2A—C2—H2B	108.1	C10—C12—H12B	109.5
O1—C3—C2	122.3 (2)	H12A—C12—H12B	109.5
O1—C3—C4	122.1 (2)	C10—C12—H12C	109.5
C2—C3—C4	115.58 (19)	H12A—C12—H12C	109.5
C14—C4—C3	112.6 (2)	H12B—C12—H12C	109.5
C14—C4—C5	113.6 (2)	C7—C13—H13A	109.5
C3—C4—C5	109.01 (18)	C7—C13—H13B	109.5
C14—C4—H4	107.1	H13A—C13—H13B	109.5
C3—C4—H4	107.1	C7—C13—H13C	109.5
C5—C4—H4	107.1	H13A—C13—H13C	109.5
N1—C5—C6	108.96 (18)	H13B—C13—H13C	109.5
N1—C5—C4	109.88 (18)	C4—C14—H14A	109.5
C6—C5—C4	111.90 (18)	C4—C14—H14B	109.5
N1—C5—H5	108.7	H14A—C14—H14B	109.5
C6—C5—H5	108.7	C4—C14—H14C	109.5
C4—C5—H5	108.7	H14A—C14—H14C	109.5
C7—C6—C5	130.0 (2)	H14B—C14—H14C	109.5
C7—C6—S2	109.81 (19)	C1—N1—C5	113.88 (17)
C5—C6—S2	120.19 (17)	C1—N1—H1A	123.1
C6—C7—N2	114.6 (2)	C5—N1—H1A	123.1
C6—C7—C13	126.8 (3)	C8—N2—C7	111.0 (2)
N2—C7—C13	118.5 (2)	C9—N3—C10	110.3 (2)
N2—C8—S2	115.3 (2)	C9—S1—C11	88.64 (14)
N2—C8—H8	122.4	C8—S2—C6	89.28 (14)
			
N1—C1—C2—C3	54.1 (2)	N3—C10—C11—S1	−0.1 (3)
C11—C1—C2—C3	175.84 (19)	C12—C10—C11—S1	177.6 (2)
C1—C2—C3—O1	127.6 (3)	N1—C1—C11—C10	−147.0 (2)
C1—C2—C3—C4	−51.0 (3)	C2—C1—C11—C10	92.3 (3)
O1—C3—C4—C14	−2.3 (3)	N1—C1—C11—S1	34.6 (3)
C2—C3—C4—C14	176.4 (2)	C2—C1—C11—S1	−86.1 (2)
O1—C3—C4—C5	−129.2 (2)	C11—C1—N1—C5	174.46 (18)
C2—C3—C4—C5	49.5 (3)	C2—C1—N1—C5	−62.4 (2)
C14—C4—C5—N1	−178.6 (2)	C6—C5—N1—C1	−174.67 (18)
C3—C4—C5—N1	−52.2 (2)	C4—C5—N1—C1	62.4 (2)
C14—C4—C5—C6	60.2 (3)	S2—C8—N2—C7	0.0 (3)
C3—C4—C5—C6	−173.35 (19)	C6—C7—N2—C8	0.6 (3)
N1—C5—C6—C7	131.6 (3)	C13—C7—N2—C8	−178.5 (3)
C4—C5—C6—C7	−106.6 (3)	S1—C9—N3—C10	−0.7 (3)
N1—C5—C6—S2	−48.5 (2)	C11—C10—N3—C9	0.5 (3)
C4—C5—C6—S2	73.2 (2)	C12—C10—N3—C9	−177.4 (3)
C5—C6—C7—N2	178.9 (2)	N3—C9—S1—C11	0.6 (2)
S2—C6—C7—N2	−0.9 (3)	C10—C11—S1—C9	−0.28 (19)
C5—C6—C7—C13	−2.1 (4)	C1—C11—S1—C9	178.4 (2)
S2—C6—C7—C13	178.0 (2)	N2—C8—S2—C6	−0.5 (3)
N3—C10—C11—C1	−178.6 (2)	C7—C6—S2—C8	0.8 (2)
C12—C10—C11—C1	−0.9 (4)	C5—C6—S2—C8	−179.1 (2)

### Hydrogen-bond geometry (Å, º)

**Table:**

*D*—H···*A*	*D*—H	H···*A*	*D*···*A*	*D*—H···*A*
C9—H9···N2^i^	0.93	2.49	3.365 (4)	157

Symmetry code: (i) *x*, −*y*+1/2, *z*+1/2.
