# Supplementary figures and images for: Crystal structure of 3-methyl-2,6-bis­(4-methyl-1,3-thia­zol-5-yl)piperidin-4-one
Source: Acta Crystallogr Sect E Struct Rep Online. 2014 Aug 30;70(Pt 9):o1055. doi: 10.1107/S1600536814018856 (PMC4186075; doi:10.1107/S1600536814018856)

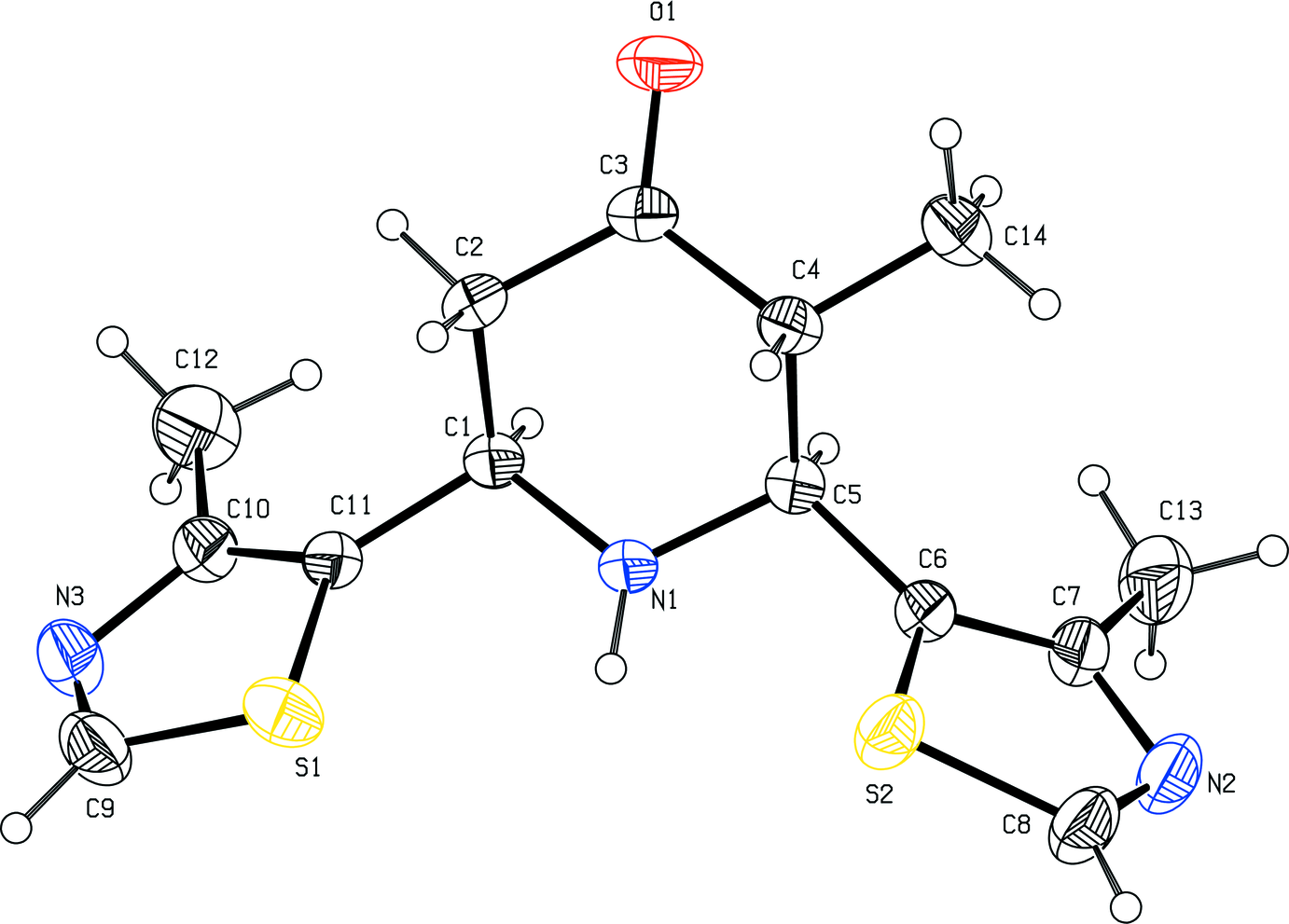

Supplement: Supplementary file 4 [file e-70-o1055-fig1.tif]

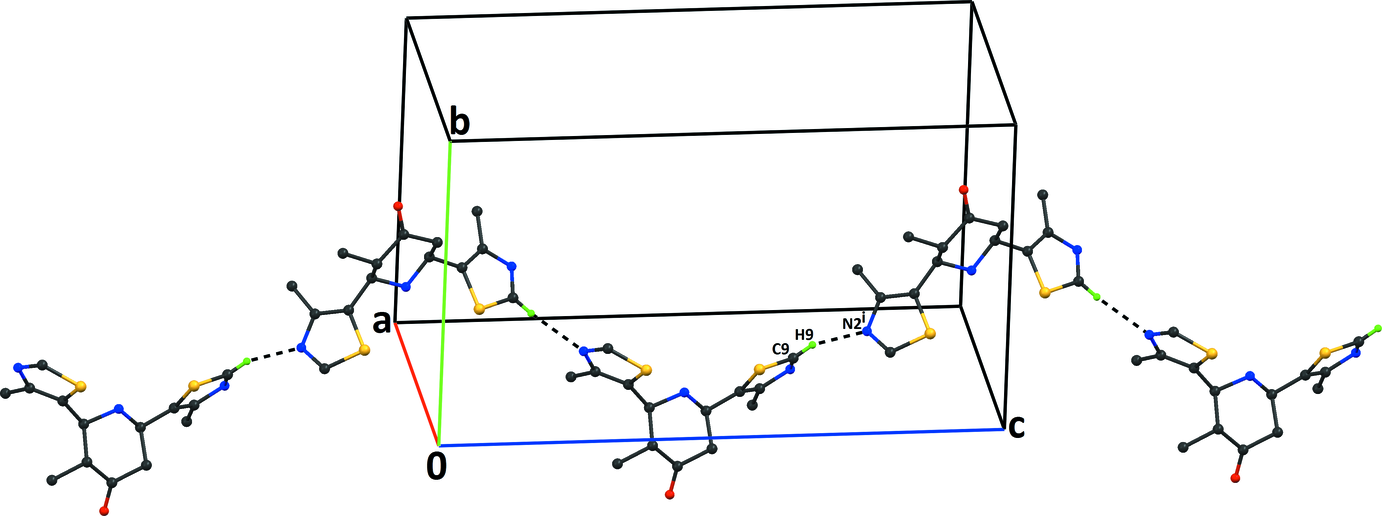

Supplement: Supplementary file 5 [file e-70-o1055-fig2.tif]
